# Impact of group educational actions on diet and quality of life of individuals with diabetes type 2

**DOI:** 10.1186/1758-5996-7-S1-A182

**Published:** 2015-11-11

**Authors:** Paula Louisy Portella Werneck, Franciele de Queiroz Santos, Marcella Lobato Dias Consoli

**Affiliations:** 1 Instituto de Ensino e Pesquisa Santa Casa De Belo Horizonte, Governador Valadares, Brazil

## Background

Diabetes mellitus (DM) is a complex chronic disease that requires continuous medical care. Nutritional therapy is part of any education program DM and contributes to the achievement of good glycemic control. The current scientific literature shows that educational interventions, especially those based on group strategies are effective on the improvement of results concerning the disease treatment.

## Objective

Evaluate through an education program the impact of group educational actions on diet and quality of life of individuals with diabetes type 2 (DM2).

## Materials and methods

This is a prospective clinical trial with 43 patients diagnosed with DM2 at least one month prior to this study. These patients were age 18 yrs. or over, users of private health care insurance. The subjects were individually evaluated before and after participation in an education program, which had five weekly meetings, lasting about an hour and 30 min each. Data collected were socio-demographic and clinical, along with weight, body mass index (BMI), waist circumference (WC), food consumption data (24-hour dietary recall – food frequency questionnaire) and quality of life (BPAID – Brazilian version of PAID scale). Shapiro Wilk test was used to evaluate normality and afterwards the T Student, Wilcoxon, Mann Whitney tests and Kruskal Wallis.

## Results

**Figure 1:**
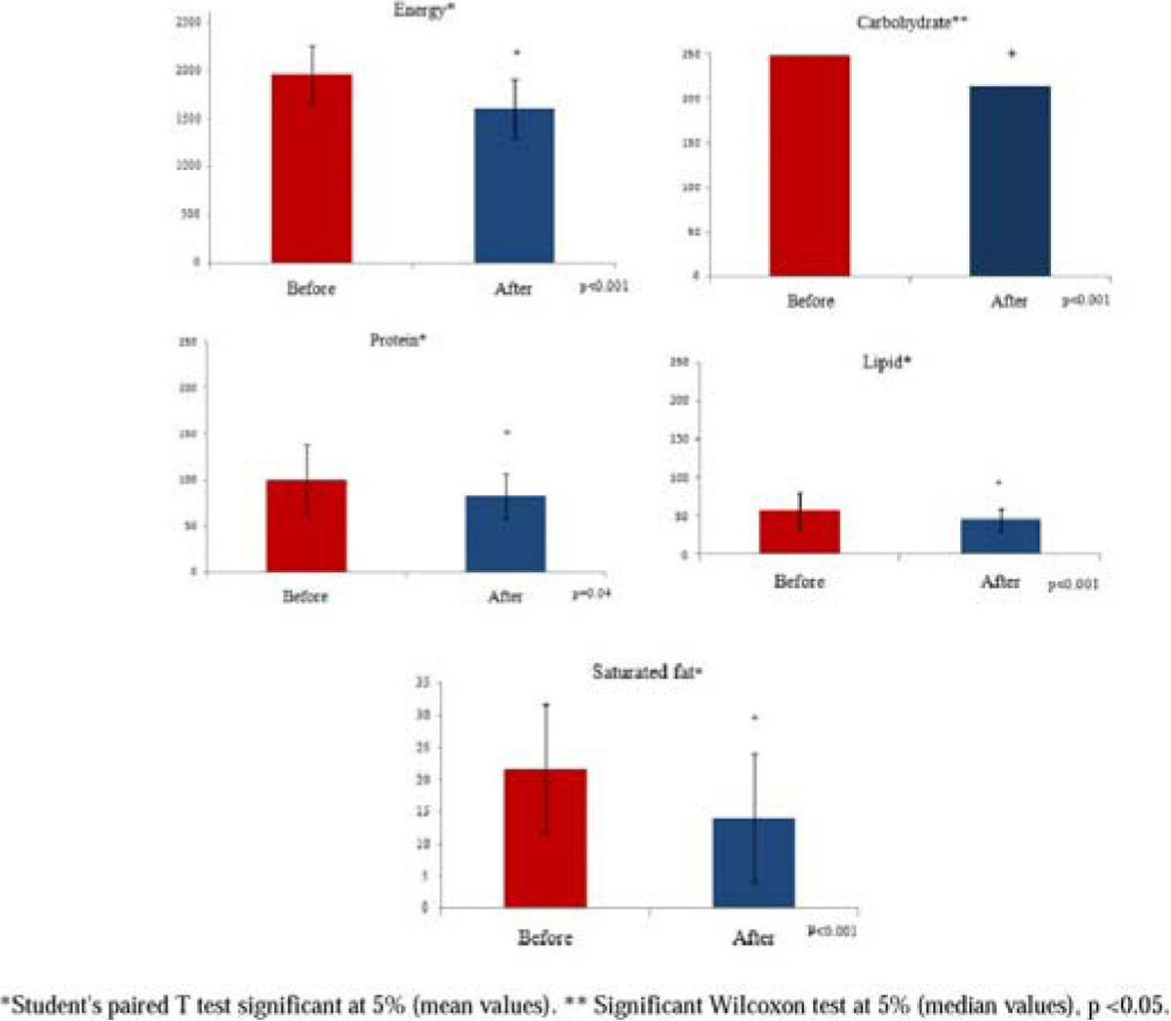
Energy consumption, carbohydrate, protein, lipid and saturated fat before and after dietary intervention in a group of 43 patients who completed the nutrition education program, Belo Horizonte, 2015.

**Figure 2:**
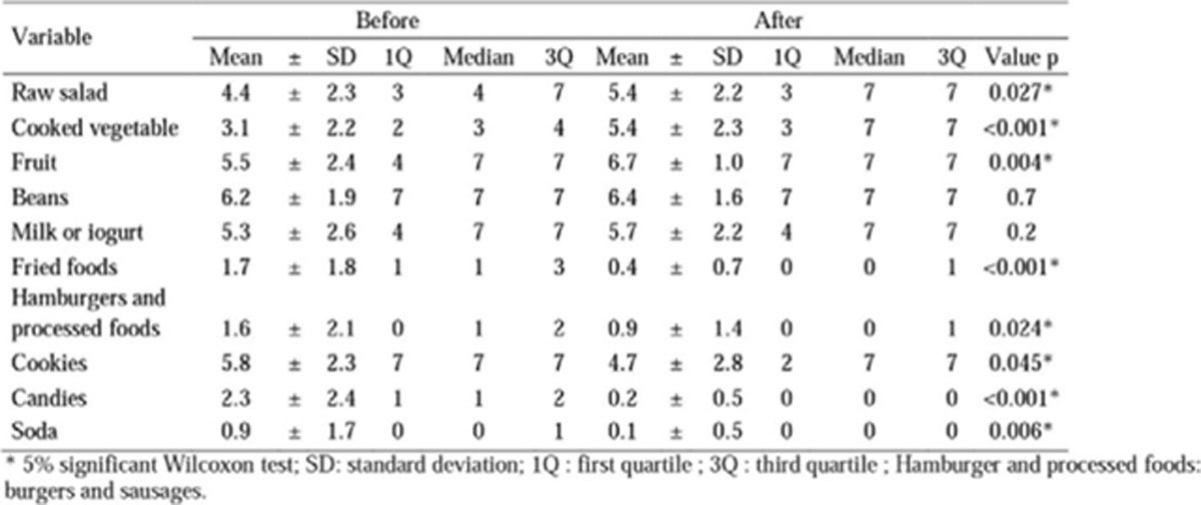
Food frequency questionnaire before and after dietary intervention in a group of 43 patients who completed the nutrition education program, Belo Horizonte, 2015.

**Figure 3:**
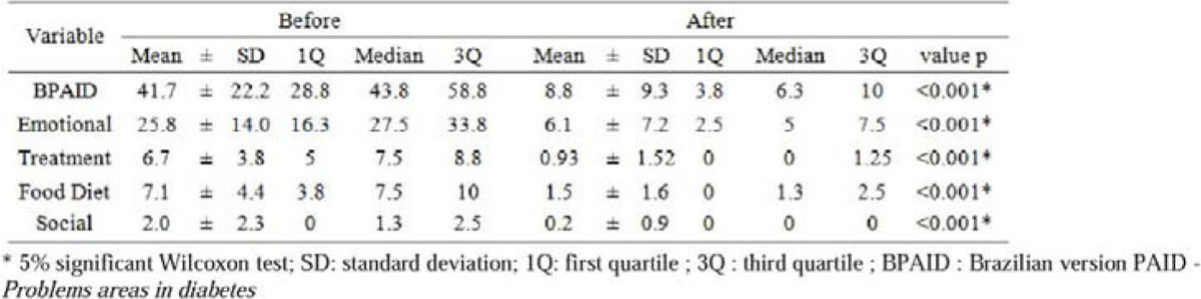
Score of BPAID scale and its sub dimensions before and after dietary intervention in a group of 43 patients who completed the nutrition education program, Belo Horizonte, 2015.

There was a significant decrease in BMI and WC (p <0.001). Nutritional assessment revealed an increase in the number of meals (p=0.006) as well as reduction energy intake, carbohydrates, proteins, lipids and saturated fat (p=0.05). The 24h recall revealed increased number of food (p=0.006), and reduction in energy intake, carbohydrates, proteins, lipids and saturated fats (p <0.05) (Figure 1). Food frequency questionnaire showed significant increase on frequency of weekly intake fruit, vegetables and salad (p<0.05). It showed a significant decrease in burgers and sausages, fried foods, cookies and crackers, candies and soft drinks (p<0.05) (Figure 2). Finally, the general score of BPAID scale showed a significant improvement in quality of life (p<0.001) after intervention (Figure 3).

## Conclusion

A nutrition intervention strictly based on education in group strategies was effective in improving participants' physical conditions, pattern of food consumption and the quality of life in relation to diabetes. This intervention model may allow for more efficient and cost-effective methods in diabetes education programs.

